# Valorizing waste streams to enhance sustainability and economics in microbial oil production

**DOI:** 10.1093/jimb/kuae041

**Published:** 2024-11-05

**Authors:** Nicholas Renegar, Seth Rhoades, Anusha Nair, Anthony J Sinskey, John P Ward, David Ross Appleton

**Affiliations:** Massachusetts Institute of Technology, Sinskey Lab, Department of Biology, Cambridge, MA 02139, USA; Fulgens Consulting, Boston, MA 02460, USA; Sime Darby Plantation Berhad, 47301 Petaling Jaya, Selangor, Malaysia; Massachusetts Institute of Technology, Sinskey Lab, Department of Biology, Cambridge, MA 02139, USA; Berry’s Brook Consulting, Rye, NH 03870, USA; Sime Darby Plantation Berhad, 47301 Petaling Jaya, Selangor, Malaysia

**Keywords:** Biofermentation, Technoeconomic analysis, Waste streams

## Abstract

Driven by the demand for more sustainable products, research and capital investment has been committed to developing microbially produced oils. While researchers have shown oleaginous yeasts and other microbes can produce low-carbon footprint oils by leveraging waste streams as energy sources, previous analyses have not fully explored the quantity of available waste streams and in turn economy-of-scale enabled on capital and operating expenses. This paper makes parallels to 2G ethanol facilities, enabling a data-driven understanding of large-scale production economics. Production costs are broken down for a variety of scenarios. The analysis finds that reaching price parity with large-scale commodity oils (e.g., palm oil, high-oleic cooking oils, biofuels feedstock oils, lauric acid) is not possible today and unlikely even under aggressive future assumptions about strain productivity. Instead, commercial production must be targeted at end markets where sustainability-conscious consumers are willing to pay the price premiums identified in this paper.

**One Sentence Summary:**

This paper makes parallels to 2G ethanol facilities, enabling a data-driven understanding of large-scale production economics for microbial lipids.

## Graphical Abstract

**Graphical Abstract:**
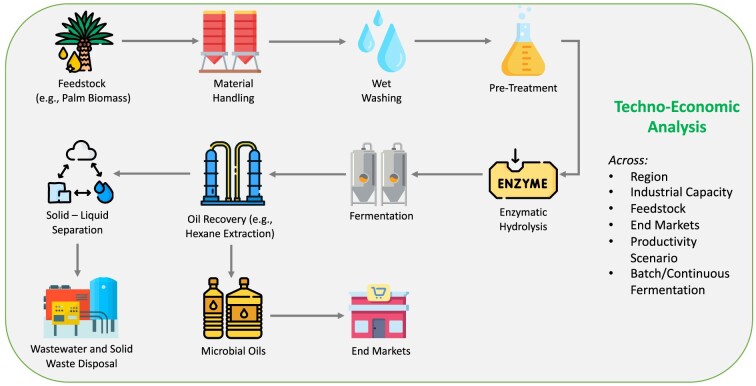

## Introduction

The growing interest in sustainable and environmentally friendly production methods has brought biofermentation technologies, particularly for lipid production, to the forefront of scientific research (Leman, 1997). This approach, which harnesses microorganisms to convert organic substrates into valuable lipids, offers a compelling alternative to traditional production methods, aligning with the global push toward environmental stewardship and circular economy principles (Masri et al., 2019).

The primary motivation behind exploring microbial lipid production is its potential to significantly mitigate the environmental impacts commonly associated with conventional lipid extraction from agricultural crops. Traditional methods have long been scrutinized for their extensive land use, deforestation implications, and high water consumption (Mattsson et al., 2000). Microbial lipid production, in contrast, utilizes renewable and often waste-derived feedstocks, presenting a solution that not only addresses environmental concerns but also contributes to carbon footprint reduction (Parsons et al., 2018).

This shift is further underscored by the increasing volume of investments flowing into the sector. A diverse range of entities are actively researching and investing in this field. These ventures are pioneering new metabolic pathways and refining biofermentation techniques to enhance yield, productivity, and cost-effectiveness, reflecting the industry’s trajectory toward leveraging biotechnology in meeting the growing global lipid demand.

However, the economic aspects of microbial lipid production, particularly its competitiveness with traditional lipid sources such as crude palm oil (CPO), remain a critical area of investigation. Koutinas et al. analyzed production of microbial oils using the yeast strain *Rhodosporidium toruloides* at an annual production capacity of 10,000T microbial oil and at zero cost of glucose, estimated a unitary production cost of $3.40/kg oil ($4.20/kg adjusted for inflation) (Koutinas et al., 2014). Braunwald et al. studied large-scale microbial production of lipids from yeast and estimated a break-even price of $2.35/kg ($3.02/kg adjusted for inflation) (Braunwald et al., 2016). Parsons et al. analyzed single cell oil (SCO) production cost from the oleaginous yeast *M. pulcherrima*, estimating a €4–8/kg breakeven sales cost depending on the feedstock (€5–9/kg adjusted for inflation) on scaling to 10,000T/year production (Parsons et al., 2018). Karamerou et al. calculated a lower-bound on cost for microbial production of lipids, estimating cost under "an ideal case that which while not achievable in reality, importantly would not be able to be improved on, irrespective of the scientific advances in this area” (Karamerou et al., 2021). Under this analysis they estimated a lower bound on production cost of $1.81/kg ($2.10/kg adjusted for inflation) at ~8,000T/year production and $1.20/kg ($1.40/kg adjusted for inflation) at ~48,000T/year production. Most recently, Caporusso et al. analyzed production costs of microbial oils by oleaginous yeasts with wheat straw as a feedstock, finding a production cost of the microbial oil of about €4/kg (Caporusso et al., 2022).

This paper presents what is believed to be one of the most rigorous and methodologically detailed techno-economic analyses to date on the feasibility of microbial fermentation as a viable long-term alternative to conventional lipid sources. The authors background in the development of commercial scale biofermentation has been combined with academic rigor to identify the salient cost elements and technical challenges in commercial-scale production. In particular, this paper develops rigorous data-driven assumptions on capital expense (by leveraging similarities between these microbial biofermentation facilities with 2G ethanol facilities as discussed in section Capital Expense and Operating FTEs), operating full-time equivalents (FTEs), feedstock costs, and other operating costs at small to large-scale production capacities. This paper also does an exhaustive review on the key cost drivers in the fermentation process, including developing distributional assumptions on theoretical yield, actual yield, and titer. Economics are presented across a range of productivity scenarios, geographic regions, feedstocks, and product targets.

## Materials and Methods

The development of a robust and comprehensive cost model for microbial lipid production required a broad set of assumptions ranging from strain productivity to operational expenses (OpEx). The initial phase in developing these assumptions involved an extensive literature review focused on understanding the distribution of productivity and titer metrics of various microbial strains employed in lipid production (see section Literature Review for full details). This review covered original research papers, scientific reviews, and meta-analyses, emphasizing studies that investigated yield optimization, strain engineering, and fermentation process efficiencies. The data extracted from these publications provided crucial benchmarks for theoretical and actual yields, titers, and growth rates.

Public data sources, including market reports, industry analyses, and financial records, were also mined to enrich the model with real-world cost parameters (see sections Capital Expense and Operating FTEs and Other Operating Expenses). This data provided a foundation for assumptions related to capital expenditure, OpEx, and revenue potential. Interviews with industry experts and stakeholders added depth to our understanding, offering qualitative insights that complemented quantitative data. Expert opinions particularly informed OpEx assumptions.

The culmination of these methodologies resulted in a dynamic cost model encapsulating key inputs across various categories, as detailed in Table 1 shown below. This model allowed for the simulation of different scenarios, reflecting variations in strain productivity, geographical regions, feedstocks, plant capacities, and product markets. Based on literature review, glucose and glycerol were modeled as biofermentation feedstocks, while corn stover, sugar beets, bread waste,^1^ cassava, and palm biomass empty fruit bunches (EFB) were modeled as additional raw material feedstocks to be converted to glucose during pre-processing. The analysis largely focuses on batch fermentation, which to our knowledge is the only process that has been successfully commercialized to date. However the impact of continuous fermentation is also modeled as it is previously demonstrated in oleaginous yeast (Abeln et al., 2020). This cost model provides a tool for examining the economic implications of diverse production setups and the feasibility of microbial lipid production under varying conditions. All costs are denoted in $USD.

**Table 1.** Cost Model Key Inputs

Category	Assumptions	Data source
Production inputs	Theoretical yield	Science literature review
Production inputs	Actual yield (as % of theoretical)	Science literature review
Production inputs	Titer	Science literature review
Production inputs	Growth rate	Science literature review
Production inputs	Recovery rate	Science literature review
Capital expense	Plant CapEx by capacity	News review of ethanol plant builds
Capital expense	CapEx multiplier by raw material	Team knowledge and scientific literature
Plant OpEx	Salaries	Team knowledge and public data sources
Plant OpEx	FTEs	Team knowledge and public 2G ethanol plant data
Production OpEx	Raw material cost	Sime Darby Plantation Berhad and public data sources
Production OpEx	Feedstock pre-processing cost	Sime Darby Plantation Berhad and public data sources
Production OpEx	Pre-processing efficiency	Scientific and technical literature
Production OpEx	Raw material yield drag	Scientific and technical literature
Production OpEx	Media cost	Team knowledge and public data sources
Production OpEx	Recovery cost	Team knowledge and public data sources
Production OpEx	Consumables cost	Team knowledge and public data sources
Production OpEx	Disposables cost	Team knowledge and public data sources
Production OpEx	Waste stream cost	Team knowledge and public data sources
Production OpEx	Facilities cost	Team knowledge and public data sources
Production OpEx	Other annual cost	Team knowledge and public data sources
Production OpEx	Tax rates by region	Public data sources
Revenue	Revenues for products	Public data sources

### Literature Review

Original research papers published within the past 15 years were assessed for their broad methodology and findings. These papers originated from a search by notable researchers and keywords related to fatty acid and oleochemical production from microorganisms. These works were in part chosen for their objectives, such as an aim to improve at least titer, yield, or productivity. Broadly, the approaches to improve the performance of these yeasts include genetic and metabolic engineering, enzyme engineering, and process optimization. The majority of organisms in these works are lipid-producing (oleaginous) yeasts, with the most common strain of yeast being *Yarrowia lipolytica*. Key measures from these papers, including strain, productivity, and yield, are listed in Table 2 below.

**Table 2.** Abbreviated Summary of Key Technical and Scientific Papers

Strain	Titer	Productivity	Yield	Lipid percentages	Source
	(g/L)	(g/L/hr)	(g/g)	(C16:0::C16:1::C18:0::C18:1::C18:2)	
*C. curvatus*	86.1	0.47	0.25	30.1::18.5::39.3::8.2	Zhang (2011)
*E. coli*	1.1	0.0153^a^	0.024	N/A	Yan (2022)
*E. coli*	1.2	0.0167^a^	0.063	N/A	Yan (2022)
*E. coli*	7.2	0.075^a^	0.049	N/A	Yan (2022)
*L. starkeyi*	17.58	0.1465^a^	N/A	36::3.5::5::47::2	Zhang (2021)
*M. pulcherrima*	26.9	0.13	0.07	17.2::4.7::0.6::69::8.4	Abeln & Chuck (2019)
*M. pulcherrima*	28.3	0.08	0.11	23.5::3.3::2.9::66.8:3.6	Abeln & Chuck (2019)
*M. pulcherrima*	44.8	0.12	0.13	30::5.1::2.3::57.7::4.8	Abeln & Chuck (2019)
*M. pulcherrima*	50.6	0.16	0.16	23.6::5.1::3.5::61.6::5.3	Abeln & Chuck (2019)
*P. putida*	0.029	0.0006^a^	0.0003	N/A	Valencia et al. (2022)
*P. putida*	0.224	0.0047^a^	N/A	N/A	Valencia et al. (2022)
*P. putida*	0.259	0.0054^a^	0.0016	13.24::18.6::0::0::0	Valencia et al. (2022)
*P. putida*	0.279	0.0058^a^	N/A	N/A	Valencia et al. (2022)
*P. putida*	0.486	0.0101^a^	N/A	10.6::10.2::0::0::0	Valencia et al. (2022)
*P. putida*	0.67	0.014^a^	N/A	5.7::7.5::0::0::0	Valencia et al. (2022)
*R. fluvialis*	23.6	0.984	0.33	29.5::0.7::14.4::35.1::14.9	Poontawee & Limtong (2020)
*R. opacus*	14.3	0.085^a^	0.279	25.7::15.8::1.8::13.8::0.9	Qiao et al. (2017)
*R. toruloides*	67.5	0.54	0.23	20::0.6::14.6::46.9::13.1	Li et al. (2007)
*R. toruloides*	78.7	0.57	0.23	29.8::1.1::5.9::53.2::5.8	Zhao et al. (2011)
*S. terricola*	13.81	0.0575^a^	N/A	29.5::1.5::8.0::56.2::2.5	Aiello et al. (2021)
*T. oleaginosus*	16.2	0.41	0.31	25::0::15.4::53.6::3.6	Meo et al. (2016)
*T. oleaginosus*	18.6	0.25	0.13	N/A	Meo et al. (2016)
*T. oleaginosus*	20.4	0.28	0.11	N/A	Meo et al. (2016)
*T. oleaginosus*	21	0.29	0.11	N/A	Meo et al. (2016)
*T. oleaginosus*	30.6	0.31	0.43	30.1::0::14.1::47.2::6.4	Meo et al. (2016)
*T. oleaginosus*	8.9	0.12	0.09	N/A	Meo et al. (2016)
*Y. lipolytica*	0.242	0.001	0.0466	N/A	Li et al. (2020)
*Y. lipolytica*	0.319	0.0012	0.0057	N/A	Li et al. (2020)
*Y. lipolytica*	1.2	0.0031	0.0964	N/A	Li et al. (2020)
*Y. lipolytica*	1.47	0.0027	0.0888	N/A	Li et al. (2020)
*Y. lipolytica*	12.5	0.1736^a^	N/A	10::25::5::30::30	Wei et al. (2021)
*Y. lipolytica*	13.5	0.1875^a^	N/A	N/A	Wei et al. (2021)
*Y. lipolytica*	2.9	N/A	N/A	N/A	Rutter et al. (2015)
*Y. lipolytica*	66.4	0.565	0.229	N/A	Xu et al. (2016)
*Y. lipolytica*	72.7	0.97	0.252	N/A	Xu et al. (2017)
*Y. lipolytica*	98.9	1.2	0.27	N/A	Qiao et al. (2017)

^a^Productivity estimated from works’ data. See Notes in corresponding spreadsheet.

In the context of commercialized chemical production, most of these works are considered early feasibility studies at a bench scale, with few exceeding 3 L. Consequently, the process optimization approaches are relatively simple, and constrained by less-sophisticated equipment. Most feeding regimens are limited to batch and simpler fed-batch of a lone carbon source, with few works harboring the equipment to control pH, dissolved oxygen, and temperature. As oleaginous yeasts are known to switch from a growth phase to a stationary, lipid-producing stage, some recent papers develop multi-stage fed-batch feeding regimens. A notable exception is the advanced semi-continuous and continuous cultivation strategies using *M. pulcherrima* (Abeln & Chuck, 2019), which motivated an added scenario to our cost modeling.

The feedstocks in these works were mostly simple sugars and first-generation feeds. Some works utilize lignocellulosic feedstocks, and many of these are motivated by a recognition that simple sugars such as glucose, while useful for R&D and strain development, are economically prohibitive, while also referencing growing examples of lignocellulosic feed utilization in bioethanol plants. The tradeoffs of these second-generation feeds are noticeable, as some key performance metrics (e.g., titer) are poor relative to glucose. This is likely due to inhibitors from the cellulosic substrate, which are not simple to address. None of these works perform direct or relative cost comparisons of alternative feedstocks to glucose, though much is referenced elsewhere and highlighted in other technoeconomic and cost modeling analyses and review papers.

**Fig. 1.**
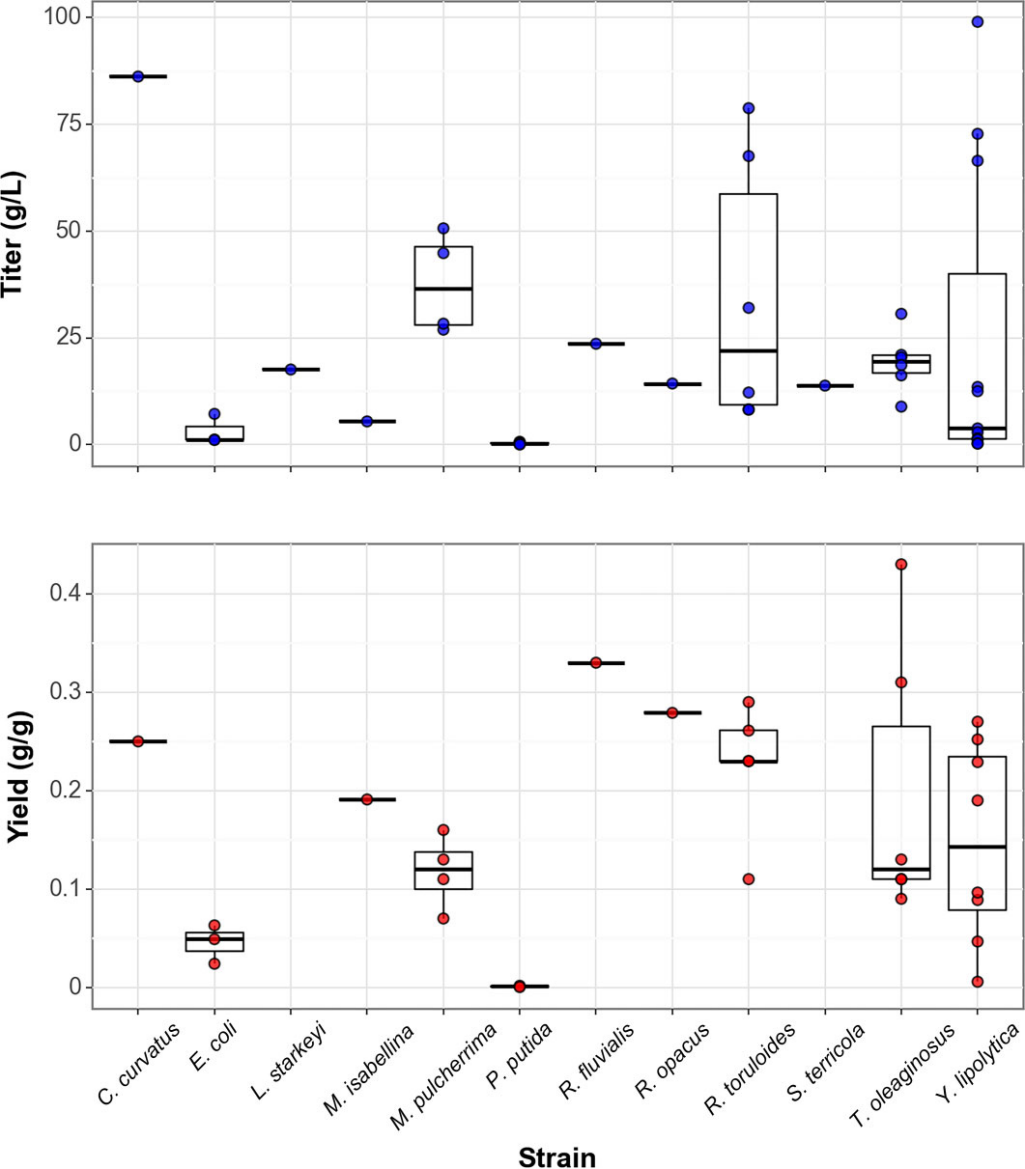
Scientific review--average performance by strain.

The full distribution of titers and yields by microbial strain is shown in Fig. 1 below. The highest reported titers and productivities across these works are approximately 100 g/L and 1.2 g/L/hr of fatty acid methyl esters in *Y. lipolytica*, fed with glucose and yeast nitrogen (Qiao et al., 2017). Titers above 30 g/L on non-standard feedstocks were reported in the lesser-studied *Trichosporon oleaginosus* strain fed with a microalgae hydrolysate (Meo et al., 2016), although the productivity was significantly lower than observed with glucose feeds in *Y. lipolytica*. Interestingly, a productivity competitive to glucose feeds was noted in an unoptimized *Rhodosporidiobolus fluvialis* strain grown on crude glycerol (Poontawee & Limtong, 2020).

The literature review was finally used to construct the various productivity scenarios for the technoeconomic analysis as follows. From these works, the highest reported titers for glucose and glycerol were 98.6 g/L and 23.6 g/L respectively, which acted as the state-of-the-art values in the technoeconomic models (noted as ‘Current Tech High’ in Table 3). The g/g lipid yields reported in these works were then used to calculate their percent of maximal theoretical yield, which for conversion of glucose and glycerol to palmitic acid are 0.35 g/g and 0.38 g/g respectively. From these numbers, best-case scenario titers were calculated, representing the theoretical maximum. These values for glucose and glycerol feeds were 111.63 g/L and 26.88 g/L respectively (‘Future Tech-High’ in Table 3). For additional comparison, the third quartile and mean of these titers were used to generate ‘Current Tech Base Case’ (57.85 g/L and 13.73 g/L, glucose and glycerol) and ‘Current Tech Low’ values respectively (32.7 g/L and 7.86 g/L, glucose and glycerol).

**Table 3.** Commodity Sales Prices and Production Costs From Biofermentation at scale in SEA (10 x 1.5M L Capacity With Palm Biomass Feedstock)

Product	Commodity	Production costs by scenario ($USD/MT)			
	Price ($USD/MT)	Current Tech Low	Current Tech Base Case	Current Tech High	Future Tech High
Crude Palm Oil	875	3,315	2,364	1,645	1,469
High-Oleic Oil	1,006	3,352	2,402	1,675	1,495
Low-CI Biofuels Feedstock Oil	1,295	3,312	2,363	1,643	1,467
75% Lauric Acid (C12)	1,120	3,588	2,533	1,753	1,566

The review additionally grounded assumptions of cycle times and plant utilization in the cost models. The smallest scale studies in this review predominantly performed batch processes of 1 L or less and averaged about 5 days of fermentation. Larger volume fermentations in this review, at upwards of 14 L, utilized a mixture of batch and fed-batch processes, and these larger fed-batch processes lasted upwards of 25 days. To form a comparable assessment of processes in the cost models, the number of batches and final performance in a given period were roughly normalized by the number of media turnovers. Turnovers of a total media volume could be estimated from fed-batch studies which reported a media dilution rate. The average dilution rate of 0.14/d in these studies corresponds to about one full volume of media exchange per week. Therefore, in comparing the reported titers possible for a given fermentation run across process types, one can consider either the titer at the end of a 5-day batch process, or that reported at the end of a month-long process, which would utilize roughly an equal volume of media. Then for simplicity in the cost models, the total number of batches producing the performance measure of interest (titer, e.g.) was drawn from the 5-day batch process scenario, which then also would generally apply to the same total output if modeled from month-long fed-batch processes.

### Capital Expense and Operating FTEs

As noted by Caporusso et al. (2022), the capital expense of building a biofermentation plant leveraging waste streams is modeled using 2G ethanol plants as a proxy. 2G ethanol plants, also known as second-generation ethanol plants, are facilities that produce ethanol fuel from non-food sources, such as agricultural waste, forest residues, energy crops, or dedicated energy crops. Unlike first-generation ethanol, which is primarily produced from food crops like corn or sugarcane, second-generation ethanol production focuses on utilizing biomass materials that are not in direct competition with the food supply.

**Fig. 2.**
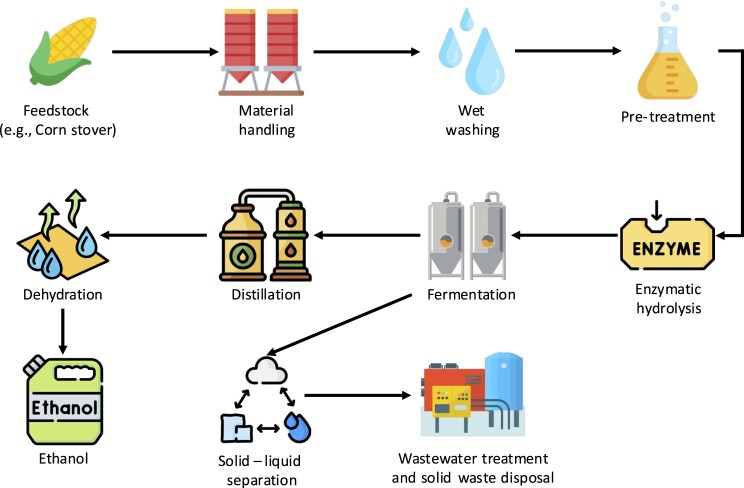
2G ethanol plant process flow.

The process of producing 2G ethanol involves the following steps, also shown in Fig. 2 (Hindustan Petrolum Corporation Limited PRAJ, 2020):

1. Feedstock collection: Biomass materials, such as corn stover are collected or cultivated.
2. Pre-treatment: The collected biomass is subjected to a mechanical, chemical, or thermal pre-treatment process, making it more accessible for further processing.
3. Enzymatic hydrolysis: The pre-treated biomass is then treated with enzymes to break down the complex carbohydrates into simple sugars, such as glucose.
4. Fermentation: The obtained sugars are fermented using specialized microorganisms, typically yeast or bacteria, which convert the sugars into ethanol through the process of anaerobic fermentation.
5. Recovery: The fermented solution is distilled to separate the ethanol from water and other impurities. Additional purification steps and dehydration may be employed to obtain a higher concentration of ethanol.

2G ethanol plants provide good benchmarks for capital expenses and operating FTEs for several reasons:

1. Similar processing steps: Both 2G ethanol production and microbial fermentation of oils involve similar processing steps, such as pre-treatment, enzymatic hydrolysis, fermentation, separation, and purification.
2. Equipment and infrastructure: Both 2G ethanol plants and microbial fermentation facilities require the same types of equipment and infrastructure. This includes fermentation vessels or tanks, separation and purification units, storage facilities, utilities, and waste treatment systems. The specific technical requirements may vary, but enough similarities exist in the core infrastructure to make meaningful cost comparisons and estimations and to drive similar economies-of-scale.
3. Experience: 2G ethanol plants have been in operation for some time and a large amount of public data is available on construction expenses and jobs created (FTEs).

While there are inherent differences between anaerobic and aerobic processes, particularly in terms of oxygen supply, agitation, and energy requirements, these cost differences largely impact operating expenses which are adjusted for separately in section Other Operating Expenses. From the authors’ commercial experience, the impact on capital expense for equipment for aeration in the aerobic process will be marginal, and furthermore capital expense itself is a relatively small small fraction of total cost-of-goods-sold at scale. As a result the reliance on 2G ethanol plants is a reasonable proxy when estimating cost-of-goods-sold, enables researchers to leverage a large amount of data on actual construction projects, and does not fundamentally alter the conclusions of the study.

**Fig. 3.**
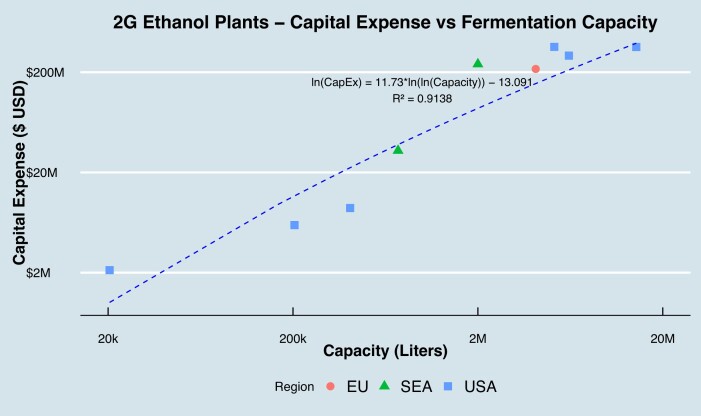
Capital investment by capacity for 2G ethanol plants.

The relationship of inflation-adjusted capital expenditures and capacity of 2G ethanol plants, based on a review of public feasibility studies, press releases on new ethanol plants, and government research studies are shown in Fig. 3 below (National Renewable Energy Laboratory, 2011). This included data points across all regions studied in the paper and commonly chosen for fermentation plant locations: USA, European Union (EU) and Southeast Asia (SEA). The coefficient of determination ($R^2$) of 0.9138 implies $91.38\%$ of the variation in capital expense can be explained by plant capacity. There is also no obvious relationship between building location and cost across the regions studied, which is expected as the majority of cost is driven by equipment and infrastructure (Hindustan Petrolum Corporation Limited PRAJ, 2020; National Renewable Energy Laboratory, 2011). In the technoeconomic analysis, capital expense’s impact on cost of goods is calculated using a 20-year depreciation.

It is further assumed that for biofermentation plants running only glucose or glycerol, a 25% decrease in equipment costs can be achieved by removing preprocessing equipment, which equates to about a 14% decrease in total capital expenditure. This amount, based on our team’s experience, closely matches the 23.6% reduction in equipment cost modeled in the NREL model when handling glucose rather than corn stover. For a biofermentation plant handling only cassava or bread waste, which just requires a grinder, blender, and hydrolysate centrifuge for pretreatment, it was assumed half of these cost savings can be achieved.

Strong correlations were also obtained for predicting plant FTEs based on capacity. The average salary and overhead for these employees was estimated at twice the average machine operator salary in that region using public datasets, to reflect the need for skilled technicians at these plants and the team’s experience in salary premium plus overhead.

Plants with fermentation capacities ranging from 50k liters (50 thousand L) up to 15M liters (15 million L) (ten 1.5Ml reactors) were selected for analysis. This upper limit is comparable to a large-scale ethanol facility as 15Ml of fermentation capacity can produce 72M gallons of ethanol per year assuming a 2-day batch time and 10% ethanol concentration in fermentation tanks. Beyond this, there are practical limitations to mixing efficiency as well as issues with material strength (particularly for concrete). Feedstock sourcing also becomes particularly challenging as you scale past this size, with 15Ml of fermentation capacity already requiring corn stover from 150,000 acres of farmland, assuming 3.75 dry tons yield per acre and our base-case productivity scenario.

### Other Operating Expenses

This section contains brief notes on all other assumptions made in the cost model. Inflation-adjusted raw material costs for glucose and glycerin were estimated at $500/MT and $600/MT respectively in the USA and EU and at $400/MT and $500/MT respectively in SEA based on literature review and public Alibaba prices, while corn stover and sugar beet were estimated at $42.50/MT and $52.70/MT respectively based on literature review (Kazi et al., 2010; Humbird et al., 2011; Liu & Chen, 2015; Aghazadeh et al., 2016; Yang & Rosentrater, 2015; Řezbová et al., 2013; Miranda et al., 2010; Shapouri & Salassi, 2006; Maung & Gustafson, 2011). Cassava and Palm Biomass (EFB) costs were estimated at $29.01/MT and $48.82/MT respectively using an internal report generated for Sime Darby Plantation Berhad and shared with the authors. Collection costs for bread waste have not been studied in detail but were estimated by the authors as twice the collection cost for palm biomass due to the relative scale of aggregation at palm mills vs. commercial bakeries. Literature review was also used to benchmark the preprocessing cost, including enzymatic hydrolysis, for converting these raw material feedstocks to glucose, which ranged from $38.10/MT for bread waste to $80.34/MT for corn stover (Humbird et al., 2011). The preprocessing efficiency (grams of glucose per gram of raw material feedstock) was also estimated based on a review of average feedstock composition from the literature (Concha Olmos and Zúñiga Hansen, 2012; Dewi et al., 2018; National Renewable Energy Laboratory, 2011). Also, critical to note is that preprocessing of these raw materials to glucose often leaves some inhibitory metabolites which impact biofermentation efficiency--which were modeled as yield drags of up to 3% based on an internal review of feedstock composition and a literature review on yield drag for ethanol production (Vanmarcke et al., 2021).

Media, consumables, and waste stream costs were estimated using NREL’s bioethanol model (National Renewable Energy Laboratory, 2011). The extraction of microbial oils typically involves intracellular lipid recovery, which may include mechanical cell disruption, solvent extraction, or combinations of both. These techniques bear similarities to algal oil extraction methods, as highlighted in the NREL report on algal oil extraction and conversion (National Renewable Energy Laboratory, 2020). While both processes involve intracellular oil recovery, the specific conditions and processing steps differ based on the microbial strains and feedstock used. We modeled the downstream recovery of lipids after fermentation with hexane extraction, as we believe this to be the most cost-effective method at scale. Extraction and solvent costs were modeled based on literature review and the team’s knowledge of commercial costs for hexane extraction of lipids from oilseeds (Parsons et al., 2018). Annual facilities expenses, including energy costs, waste treatment and maintenance, were modeled at 5.0% of capital expense based on the team’s experience, closely matching the 5.45% from the NREL bioethanol model (National Renewable Energy Laboratory, 2011). Other annual expenses, including insurance, compliance, and legal costs were estimated at 16.9% of facilities annual costs based on the NREL bioethanol model (National Renewable Energy Laboratory, 2011). Corporate tax rates for the US, EU, and SEA were based on averages for those regions.

### Product Revenue

**Fig. 4.**
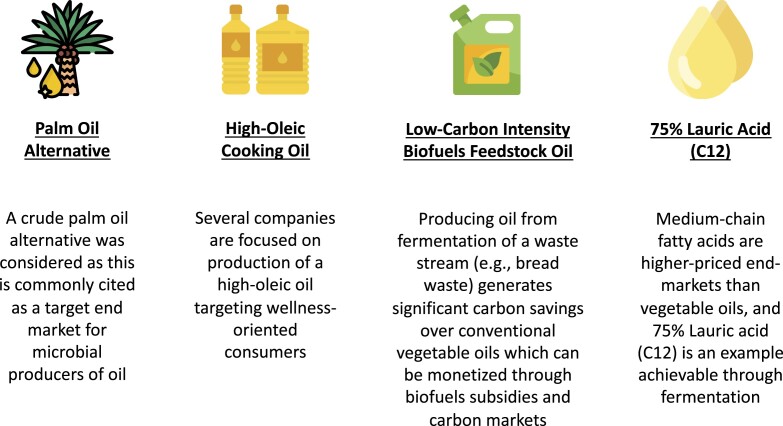
The four end-markets analyzed.

Four products were included in the cost model, with the motivation for studying each end market shown in Fig. 4 below. All four markets are large enough that price elasticity curves are not relevant at the volumes achievable at a single large-scale biofermentation facility and considered in this paper.

For the CPO alternative, revenue was estimated at $875/MT based on daily settlement prices available from the Malaysian Palm Oil Council. For the high-oleic cooking oil, such oils when conventionally produced can receive a 20% price premium over conventional vegetable oils in commodity markets, giving a revenue of $1,006/MT. When using microbial oils as a low-carbon intensity biofuels feedstock oil, the carbon saving premiums can be directly monetized in a transparent way. Prices were estimated based on offtake by US biofuels producers, and using soybean oil spot prices from Chicago Board of Trade as well as public credit prices from California’s Low Carbon Fuel Standard Program minus transportation costs by super-liner to the nearest US port. These revenues were estimated at $1,389/MT for microbial oil production in the US, $1,307/MT for production in the EU, and $1,295/MT for production in SEA. More information on these carbon subsidies can be found in Appendix A. For the 75% Lauric Acid (C12), revenue was estimated at $1,120/MT based on public data from Alibaba.

## Results and Discussion

Technoeconomic analysis was done to analyze production costs of microbial oils from oleaginous yeasts, covering over 2,000 scenarios across batch and continuous fermentation, different productivity levels, fermentation capacities, regions, raw material feedstocks, and end-markets (see Section Materials and Methods for a full list of evaluated scenarios). Key results and strategic insights across these many scenarios are shared in the following sections, with all costs denoted in $USD.

### Production Cost by Capacity, Region, and Feedstock

**Fig. 5.**
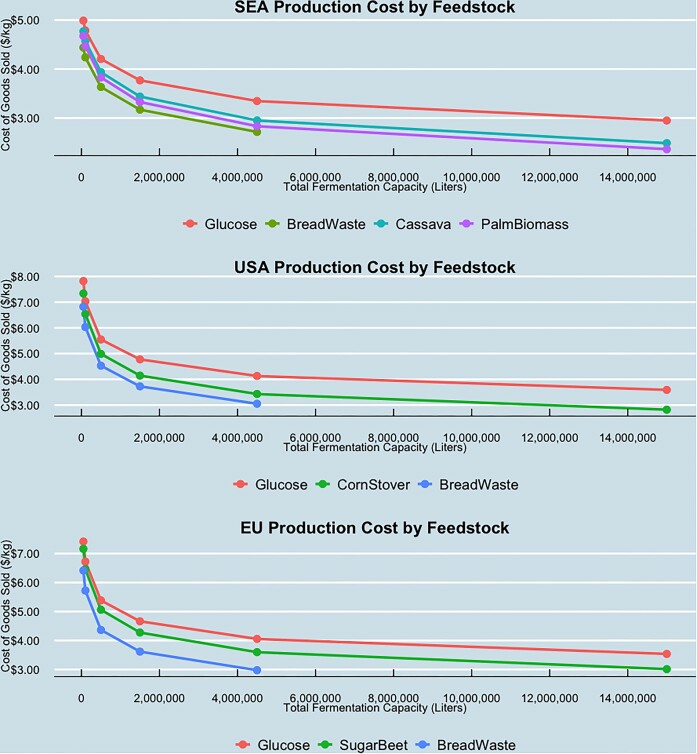
Palm oil alternative production cost by feedstock and region. (Current Tech - Base Case Productivity Scenario).

Fig. 5 below shows the estimated production cost by region, feedstock, and plant capacity for a palm oil alternative produced from batch fermentation of glucose under our base case productivity scenario. Feedstocks were only modeled for regions where they would be available: cassava and palm biomass feedstocks for SEA, corn stover for the USA, and sugar beet for the EU. Additionally, production costs for bread waste are only modeled for plant capacities up to 4.5M L; a decision based on our internal assessment of feedstock availability from commercial bakeries.

We note that there is significant economy-of-scale as plant capacities increase, although these cost savings taper off at the upper limits of feasible fermentation capacity. For production of a palm oil alternative from glucose in the USA, production costs range from $7.82/kg at 50k L to $3.59/kg at 15M L. We also observe that production costs in the EU are slightly lower than the USA, while costs in SEA are significantly lower due to reduced labor and feedstock costs. For production of a palm oil alternative from glucose in the SEA, production costs range from $4.99/kg at 50k L to $2.95/kg at 15M L.

Fig. 5 also shows the economic need for waste stream valorization in microbial fermentation. Across all regions, waste streams can significantly reduce production costs relative to glucose. For production of a palm oil alternative from corn stover in the USA, production costs range from $7.38/kg at 50k L to $2.82/kg at 15M L. The lowest production costs can be achieved in SEA, with cassava allowing for a production at $2.49/kg at 15M L of fermentation capacity and palm biomass (EFB) allowing a production cost of $2.36/kg at 15M L.

Bread waste is the lowest production cost feedstock at all given capacity levels due to pre-fermentation conversion efficiency (grams of glucose per gram of feedstock), as well as the reduced capital expenditures enabled by simpler feedstock pre-processing. While corn stover in the USA and cassava and palm biomass in SEA can offer better production cost due to the additional feedstock availability and scale, bread waste in the EU at 4.5M L of fermentation capacity equates to the lowest production cost at $2.98/kg, beating out production from sugar beets at increased scale.

### Breakdown of Production Cost Drivers

**Fig. 6.**
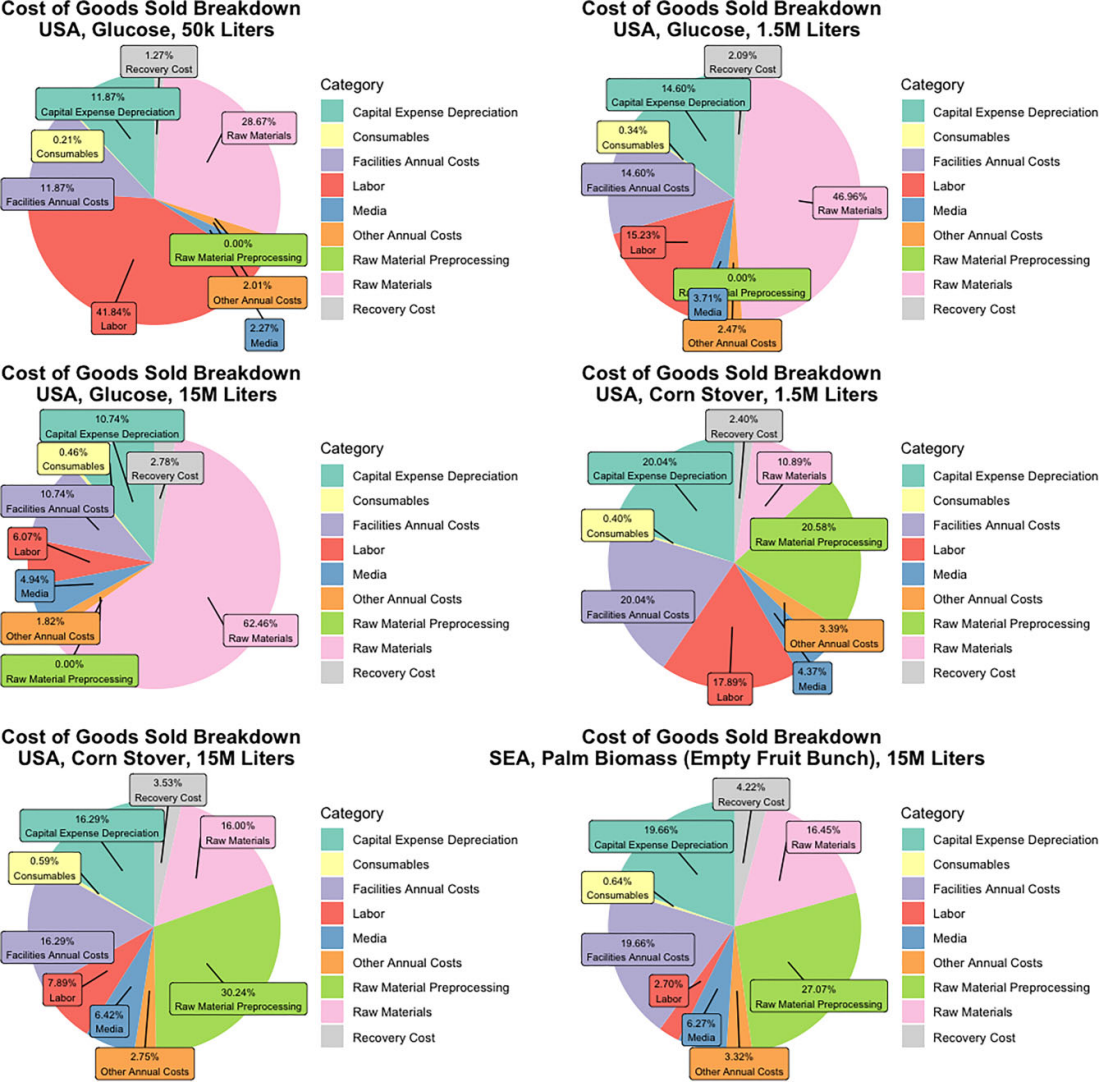
Cost of goods sold breakdown by capacity, feedstock, region for a Palm Oil alternative.

Fig. 6 shows a breakdown of cost drivers for microbial production of a palm alternative for six instances modeled in this technoeconomic analysis under the base-case productivity scenario. These scenarios were selected as representative of the major key insights into production costs.

At small scale production (50k L of fermentation capacity) from glucose in the USA, labor is the largest cost percentage at 41.84%, although there is a significant economy-of-scale on fermentation capacity vs. FTEs such that labor represents only 15.23% of the production cost at 1.5M L of fermentation capacity and 6.07% at 15M L of fermentation capacity. Although capital expenditure to build the facility also offers economy-of-scale and is monotonically decreasing when expressed as a $/kg cost as scale increases, its percentage of the total spend does not monotonically or significantly decrease due to the drastically better economy-of-scale on labor. For production from glucose in the USA we have plant capital expense depreciation (on a 20-year time horizon) representing 11.87% of cost of goods sold at 50k L, 14.60% of cost of goods sold at 1.5M L, and 10.74% cost of goods sold at 15M L. In general, as these fermentation capacities change from small-scale commercial to large-scale commercial, we observe that cost of goods sold goes from being dominated by labor and capital expenses, to being dominated by raw material cost, with glucose representing 62.46% of production cost at 15M L capacity.

To understand the impact of waste stream valorization on cost, comparisons were made between glucose and corn stover feedstocks at 1.5M and 15M L. In both instances, using a waste stream decreases the total raw materials and raw material processing cost, while increasing capital expenses and facilities costs (including energy). For production from corn stover at 15M L of fermentation capacity, 46.24% of the total production cost is driven by feedstock and preprocessing costs, while the remaining cost is spread broadly across capital expense, facilities, labor, media, and recovery costs.

We pay special attention to the final scenario, which is production from palm biomass (EFB) in SEA at 15M L of fermentation capacity. This lowest cost scenario from the section Production Cost by Capacity, Region, and Feedstock. We see that labor is a tiny fraction of the total production cost at 2.70%, while raw materials represent 16.45%, pre-processing costs are 27.07%, and capital expense and facilities costs are both 19.66%. This relatively even distribution of cost drivers implies that no external price changes (e.g., decreases in steel costs for plant construction or labor costs) could significantly reduce production costs on its own.

### Future Potential Cost Reductions Through Strain and Technology Improvement

Various productivity scenarios were studied, as described in section Literature Review. We find that based on the literature review of current production technology and yields produced at bench scale, we can conservatively upper bound the production cost at $3.31/kg (‘Current Tech Low’) and lower bound the production cost at $1.64/kg (‘Current Tech High’) at scale. These scenarios represent typical bench-scale titers and best-in-class bench-scale titers, respectively. Under our most aggressive scenario about future strain productivity, beyond anything that has been achieved at bench-scale today, it is projected that production costs could reach as low as $1.47/kg.

### Gross Margins by End Market

Table 3 below shows the commodity sales prices and production costs from biofermentation at scale in Southeast Asia (10 x 1.5 million-liter capacity with palm biomass feedstock) for the four end markets described in section Product Revenue. While sections Production Cost by Capacity, Region, and Feedstock and Breakdown of Production Cost Drivers detailed production costs for a palm oil alternative, it is critical to note that production costs are similar across all of these end markets due to similar theoretical strain yields (grams of final product per gram of glucose) across all fatty acid profiles. In particular, after strain optimization to reach the desired lipid fatty acid profile with a similar titer and actual yield, production costs vary by just a couple of percentage points.

We see from Table 3 that even under extremely aggressive future productivity assumptions—including a higher titer than ever achieved at bench scale across our broad literature review and 100% actual yield—production costs remain significantly higher than the commodity prices for all products considered. The gross margins, calculated as $(\text{Revenue} - \text{Cost of Goods Sold}) / \text{Revenue} * 100\%$, remain negative across all scenarios and products.

Specifically, the gross margins for each product range as follows:

1. **Crude Palm Oil**: Gross margins range from $-278.8\%$ under the Current Tech Low scenario to $-67.9\%$ under the Future Tech High scenario.
2. **High-Oleic Oil**: Gross margins range from $-233.2\%$ to $-48.6\%$.
3. **Low-CI Biofuels Feedstock Oil**: Gross margins range from $-155.8\%$ to $-13.3\%$.
4. **75% Lauric Acid (C12)**: Gross margins range from $-220.4\%$ to $-39.8\%$.

This indicates that even when commercial biofermentation of microbial oils reaches the scale of 2G ethanol production, true price competitiveness with commodity vegetable oils is unlikely to be achieved based on today’s prices. Moreover, combining this with the insights from Section 3.2, we see that a broad set of underlying fundamental shifts would be required for price parity. No individual cost driver is significant enough to allow price parity.

The negative gross margins above only hold when selling into these commodity markets at prevailing commodity prices. There may be an opportunity to leverage the sustainability factor for additional pricing power. Of the end markets studied, only using microbial oils as a low-CI biofuels feedstock directly monetizes the land use and carbon savings. While current transportation decarbonization programs do not seem to pay enough to support commercial success, there could be better opportunities in the consumer products space. In these markets, the higher production cost of microbial oils can be economically justified by consumers’ increasing willingness to spend on healthier and more sustainable food or green cosmetics (Sharma et al., 2021).

### Potential Impact of Continuous Fermentation

We also modeled the impact of continuous or semi-continuous fermentation as this could be enabled by certain oleaginous yeast strains such as *M. Pulcherrima* (Abeln et al., 2020), although to our knowledge only batch fermentation has been successfully used in commercial biofermentation. While continuous production could enable better economics due to higher feedstock throughput and less media turnover relative to fermentation capacity, our technoeconomic analysis found that it is unlikely to significantly shift economics. In particular, for the lowest production cost scenario of microbial oil production from palm biomass in SEA at 15M L of fermentation capacity, we modeled that continuous fermentation could reduce production costs from $2.36/kg to $2.25/kg. This production cost decrease of 4.7% is not material enough to change any of the previous conclusions.

### Strategic Insights

The key strategic insights are summarized below:

1. Strong economy-of-scale exists on production costs, with the cost of producing microbial oils produced in the USA using glucose decreasing from $7.82/kg at 50k L of fermentation capacity to $3.59/kg at 15M L of fermentation capacity.
2. Valorizing waste streams is critical for lowering costs across all regions studied. Microbial oil production costs in the USA can be as low as $2.82/kg at 15M L of fermentation capacity by using corn stover, production costs in Southeast Asia can be as low as $2.36/kg at 15M L of fermentation capacity by using palm biomass (EFB), and production costs in the EU can be as low as $2.97/kg at 4.5M L of fermentation capacity by using bread waste. Under aggressive assumptions about future strain productivity, it is projected that production cost could reach as little as $1.47/kg under today’s prices.
3. Continuous fermentation could lower costs if it were successfully operationalxdized, but not by a material amount.
4. When selling into commodity end markets, we find that gross margins are negative even under aggressive assumptions about potential future strain productivity and technology. This implies a need to sell into consumer markets where the sustainability benefits can be directly monetized to enable attractive unit economics.

## Conclusion

Technoeconomic analysis was performed to study the production costs of microbial oils across a variety of regions, fermentation capacities, feedstocks, end markets, and productivity scenarios. It was found that production costs as low as USD$2.36/kg could be enabled at scale through waste stream valorization in Southeast Asia; however, this would still yield negative margins for the broad range of accessible commodity end-markets studied, including using the microbial oil as a low-carbon intensity biofuels feedstock oil to monetize the land-use and decarbonization impact. This implies a need to sell into consumer markets where the sustainability benefits can be directly monetized to enable attractive unit economics.

Future work should explore strategies to further reduce production costs and improve economic viability. One potential avenue is the implementation of fed-batch high cell density cultivation, which could enhance productivity by increasing cell concentrations and lipid yields. While this approach presents challenges in terms of scalability and operational complexity, successful research and operation in this area could lower costs and improve the competitiveness of microbial lipid production. Additionally, advancements in strain engineering to improve yields and titers, optimization of downstream processing techniques, and innovations in fermentation technology—such as continuous fermentation or novel bioreactor designs—could contribute to cost reductions. Exploring these strategies could help bridge the gap toward achieving price parity with commodity vegetable oils and expand the applicability of microbial oils in various markets.

By addressing these technological and operational challenges, there is potential to make microbial lipid production more economically feasible. Continued research and development in these areas will be critical for the future success of microbial oils as sustainable alternatives in both commodity and specialty markets.

## Data Availability

The data assumptions used for this study are available in a public GitHub repository. The data include assumptions related to capital and operating expenditures, waste stream availability, microbial productivity, and input costs used in the analysis. The repository can be accessed at: https://github.com/nrenegar/JIMB-Valorizing-Waste-Streams.

## Footnotes

1 Bread waste refers to surplus or discarded bread that isn’t suitable for sale or consumption due to reasons like overproduction, expiration, or spoilage.

## Appendix A. Biofuels feedstock markets

A low-carbon biofuels feedstock oil was chosen for analysis to best understand the potential of microbial fermentation to displace palm oil in a large commodity end-market. Palm oil is commonly used as a biodiesel feedstock in many countries today, and an additional 6 billion gallons of US biofuels production is being built by 2025 that could provide an excess of demand for low-carbon microbial oils.^2^ This market will also enable better pricing than replacing crude palm oil, due to the increased carbon subsidies that could be accessed.

For revenue, pricing was determined based on substituting the microbially produced oil for refined, bleached, and deodorized (RBD) soybean oil in US biofuels production. The microbially-produced oil can act as a drop-in replacement for soybean oil because of its similar fatty acid profile, and like crude soybean oil would require similar RBD refining costs to get negligible levels of phosphorous and other heavy metals and overall specs compatible with biofuels catalysts. Therefore, we benchmarked the price based on Chicago Mercantile Exchange’s CBOT crude Soybean oil spot price^3^ along with revenue sharing for the carbon subsidy premium over soybean oil.

In the US, carbon savings are monetized in several ways. First, there are flat subsidies for biofuels provided they meet at least a certain carbon savings threshold, including the biodiesel production and blending tax credit and Environmental Protection Agency’s Renewable Fuel Standard RINS credits. For these subsidies, soybean already has high enough carbon savings relative to petroleum to earn these per-gallon subsidies, so there is no value premium for a lower-carbon feedstock oil. Second, there are also programs with carbon subsidy amounts proportional to the amount of carbon savings such as California’s Low Carbon Fuel Standard and the Clean Fuel Producer Tax Credit, where additional carbon intensity (CI) savings can be directly monetized. Based on these credits, and assuming a CI score of 17 for the microbial oil based on a literature review and industry discussions, we calculated a carbon premium of $0.508 per gallon of finished biodiesel (Parsons et al., 2018). Assuming half of the carbon premium accrues to the low-CI feedstock producer, this implies a microbial oil produced in the US could command $1,389/ton as a biofuels feedstock source. Based on the additional transportation costs to the US, the revenue commanded could also be as much as $1,307/ton in the EU and $1,295/ton in Southeast Asia.
